# Calcifediol Is Not Superior to Cholecalciferol in Improving Vitamin D Status in Postmenopausal Women

**DOI:** 10.1002/jbmr.4560

**Published:** 2022-05-06

**Authors:** Manuel Sosa‐Henríquez, M.a Jesús Gómez de Tejada‐Romero, M.a Jesús Cancelo‐Hidalgo, Guillermo Martínez Díaz‐Guerra, Íñigo Etxebarría Foronda, Francisco José Tarazona‐Santabalbina, Óscar Torregrosa‐Suau, Carmen Valdés‐Llorca

**Affiliations:** ^1^ University of Las Palmas de Gran Canaria, Investigation Group on Osteoporosis and Bone and Mineral Diseases Las Palmas de Gran Canaria Spain; ^2^ Bone Metabolic Unit Hospital University Insular Las Palmas de Gran Canaria Spain; ^3^ Department of Medicine University of Seville Seville Spain; ^4^ University Hospital of Guadalajara Alcalá University Madrid Spain; ^5^ University Hospital 12 de Octubre. Madrid University Complutense Madrid Spain; ^6^ Department of Orthopaedic Alto Deba Hospital Mondragon Spain; ^7^ Geriatric Service University Hospital La Ribera Alzira Spain; ^8^ Bone Metabolic Unit. Service of Internal Medicine University General Hospital Elche Spain; ^9^ Health Center of Fuencarral. SERMAS Madrid Spain

To the Editor:

We have read with interest the article published in the *Journal of Bone and Mineral Research* (the *JBMR*) by Pérez Castrillón and colleagues,^(^ ^1^ ^)^ comparing the efficacy and safety of calcifediol versus cholecalciferol in improving vitamin D status in postmenopausal women.

We would point out a series of innacuracies that question the validity and certainty of their conclusions. Due to limitations we cannot cite them all, but we describe the most relevant:

- The women studied are postmenopausal with hypovitaminosis or vitamin D deficiency (25(OH) vitamin D levels less than 20 ng/mL). Therefore, the results cannot be extrapolated to all postmenopausal women, as the title suggests.
- The cholecalciferol doses prescribed are not those recommended for subjects with vitamin D deficiency. The authors justify the monthly dose of cholecalciferol (25,000 IU) recommended by Kanis and colleagues^(^ ^2^ ^)^ and Pludowski and colleagues.^(^ ^3^ ^)^ However, in Kanis and colleagues’^(^ ^2^ ^)^ guidelines, the doses are for treating osteoporosis, not for vitamin D deficiency, so not applicable here. Furthermore, the Pludowski and colleagues^(^ ^3^ ^)^ guidelines indicate that “for patients with a laboratory confirmed vitamin D deficiency, ie, 25(OH)D concentration lower than 20 ng/mL (50 nmol/L), a vitamin D treatment should be implemented. (…). The dosage should be as follows (…): for adults and the elderly 7000–10,000 IU/ day (175–250 mg/day) or 50,000 IU/week (1250 mg/week).” Clearly, the cholecalciferol dose was not adequate, but markedly lower than those recommended in this latest, reported guideline. Therefore, the cholecalciferol treatment group was underdosed.

Other guidelines recommend that, regarding vitamin D deficiency, defined by levels of 25(OH) vitamin D below 20 ng/mL, higher doses than those indicated here should be prescribed. The Endocrine Society recommends cholecalciferol doses administered at 50,000 IU weekly for 8 weeks (alternatively 6000 IU daily), followed by 1500–2000 daily maintenance IU^(^ ^4^ ^)^; the National Osteoporosis Society recommends 2000 IU daily,^(^ ^5^ ^)^ between 45,000 and 60,000 IU monthly of cholecalciferol. More recently, the American Association of Clinical Endocrinologists (AACE) recommended 5000 IU daily for 8 to 12 weeks^(^ ^6^ ^)^; ie, 150,000 IU monthly.

Thus, 25,000 IU of cholecalciferol administration once a month used by Pérez Castrillón and colleagues^(^ ^1^ ^)^ is insufficient and explains why they deem cholecalciferol “inferior” to calcifediol.

- The article reflects partial results at 4 months in a study designed for 1 year. This should have been reflected in the title. According to the reported dates, the last patient would have completed his annual visit on June 25, 2020 (visit at 4 months: October 25, 2019). The article with the results at 4 months was sent to the journal in February 2021. What happened in those remaining 8 months? Why have those data not been shown?

It is important to know the percentage of patients who, in this 4–12‐month window, develop 25(OH) vitamin D levels above the optimal desirable range of 30–50 ng/mL.^(^ ^7^ ^)^ It would be interesting to know the speed with which the levels of 25(OH) vitamin D fall again after discontinuing calcifediol at 4 months (group A.2). The authors have not included a similar group of cholecalciferol that would permit comparisons. Also, not all clinically relevant results were taken into account, because although it is important to correct low vitamin D levels, the ultimate benefit is to prevent hypovitaminosis complications. The time in which vitamin D levels remain stable after treatment is not specified; it only focuses on correcting levels and the speed with which correction occurs. A quick correction would not be useful if after 4 months complications begin to appear. The expected benefits do not currently outweigh the risks and costs, because the long‐term adverse effects of calcifediol are not known and cholecalciferol treatment has so far been effective, safe, and cheap.^(^ ^8^, ^9^ ^)^

In our opinion, this study of Pérez Castrillón and colleagues^(^ ^1^ ^)^ holds some issues of concern that could invalidate the results shown and could mislead readers to a false understanding.

## Author Contributions

**Manuel Sosa‐Henríquez:** Conceptualization; investigation; methodology; supervision; validation; visualization; writing – original draft; writing – review and editing. **M.a Jesús Gómez de Tejada‐Romero:** Conceptualization; methodology; supervision; validation; visualization; writing – original draft. **M.a Jesús Cancelo‐Hidalgo:** Conceptualization; methodology; supervision; validation; visualization. **Guillermo Martínez Díaz‐Guerra:** Conceptualization; supervision; validation; visualization. **Íñigo Etxebarría Foronda:** Conceptualization; investigation; supervision; validation; visualization. **Francisco José Tarazona‐Santabalbina.:** Conceptualization; supervision; validation; visualization. **Óscar Torregrosa‐Suau:** Conceptualization; methodology; supervision; validation; visualization. **Carmen Valdés‐Llorca:** Conceptualization; investigation; resources; supervision; validation; visualization.
